# A short note on oxytocin and stress attenuation

**DOI:** 10.6026/97320630017921

**Published:** 2021-11-30

**Authors:** Tamilselvan Kuppusamy, Padmavathi Ramaswamy, Muraliswaran Perumal, Santhi Silambanan, Archana Prabu Kumar

**Affiliations:** 1 Sri Venkateshwaraa Medical College Hospital & Research Centre, Puducherry, India; 2 Sri Ramachandra Medical College and Research Institute, SRIHER, Chennai, India; 3 College of Medicine and Medical Sciences, Arabian Gulf University, Manama, Bahrain

**Keywords:** stress, allostasis, allostatic overload, oxytocin, stress attenuation

## Abstract

Stress is integral part of life and it initiates appropriate response at times of adversities to promise survival. Stress could be either physiological or psychogenic. Stress is often psychogenic in nature and it induces the release of cortisol from adrenal cortex into circulation by activating Hypo thalamo-pituitary-adrenal axis (HPA). Cortisol thus released mediates the stress response by its catabolic effects to enhance the activity of vital organs during emergency. However, prolonged activation of the HPA axis can lead to physical and mental illness as an outcome of persistent stress. Nature has bestowed the biological system with an array of endogenous mechanisms to buffer stress. Oxytocin, a nano-peptide released by the magno-cellular neurons of hypothalamic paraventricular nucleus (PVN) is an efficient stress buffering neuro-peptide. This hormone mediates many physiological and behavioural functions get released during stress. It attenuates the stress axis initiated by the release of corticotropin releasing hormone (CRH) from the parvocellular neurons of the same hypothalamic nucleus. Oxytocin released by PVN exerts an inhibitory effect on the release of CRH by down-regulating the expression of the gene that transcribes for this hypothalamic hormone. Thus, it inhibits the release of adreno cotico trophic hormone (ACTH) and cortisol, exerting an overall suppressive modulation of the stress axis and attenuates stress.

## Background:

Oxytocin, the neuropeptide of central nervous system, mediates many physiological and behavioural functions. The stress buffering capacity is among the less explored functions of oxytocin. Therefore, it is of interest to document a short note on oxytocin and stress attenuation.

## Stress, stress response and stress related disorders:

The constancy of the internal environment is critical for the maintenance of life. Any factor, either physiological or psychogenic, that seriously threatens the homeostasis disturbs the stability of internal environment. The outcome of such a disturbance imposed on the biological system is called "stress" and in response to stress the organism displays a coping response with adaptive changes in cardiovascular, metabolic and immune system functioning, mediated by sympathetic nervous system arousal and activation of hypo thalamo pituitary adrenal axis [1]. The spectrum of beneficial adaptations achieved to establish homeostatic stability is called allostasis. These adaptive changes can subject the biological system to wear and tear and sometimes adaptation failure results leading to allostatic overload signifying the burden of stress on the biological system [2]. It has been observed that maladapted stress response can predispose to a wide range of pathologies like hypertension, coronary artery calcification, obesity and the risk of developing obesity with symptoms of depression and anxiety, cancer, externalizing, internalizing, fatigue, inflammatory/immune response, other mental health and physical health problems [1].

## Stress activates HPA axis:

Physiological stress stimulates Paraventricular Nucleus (PVN) by direct noradrenergic or peptidergic stimulation, whereas psychogenic or anticipatory stress involves disinhibition of PVN by trans-synaptic inhibition of tonic GABAergic activity in the immediate surround of PVN by amygdala.The disinhibition of corticotropin releasing hormone (CRH) producing parvocellular cells of PVN causes the release of CRH that initiates the stress axis by having increased CRH gene transcription via a cAMP/PKA-dependent mechanism [3]. The transcription begins when an activator of the gene namely cAMP-responsive element binding (CREB) protein gets phosphorylated by cAMP and binds to a cAMP-responsive element (CRE) in the promoter region accompanied with the binding of a CREB coactivator, CREB-regulated transcriptional coactivators (CRTC1-3) to CREB. In basal conditions these CRTC remains phosphorylated and are bound to the scaffolding protein 14-3-3 in the cytoplasm which then translocates to the nucleus on dephosphorylation, where it binds CREB with its bZIP domain that helps in recruitment of CREB Binding Protein (CBP/p300) to the CRH promoter for gene transcription, by its coactivation [4].

The corticotropin releasing hormone (CRH) thus released, on reaching the corticotropes of anterior pituitary induces the release of ACTH into the systemic circulation, by enhancing the transcription of the pro opiomelanocortin (POMC) gene, which encodes for it. The circulating ACTH on binding to its receptors on the adrenal cortex stimulates the synthesis and secretion of cortisol that mediates stress adaptive response by its interaction with the intracellular receptors in the target cells [5]. The HPA axis is accentuated by nor adrenergic stimulation from Locus coeruleus (LC) and Nucleus tractus solitarius (NTS) with the former being driven by amygdala-hippocampal systems and the latter by afferent sensory stimuli [6]. When stress response gets out of control its ill effects shall outweigh the benefits thus shifting allostasis to allostatic overload.

## Oxytocin, an efficient stress attenuator:

In response to stress, oxytocin, a nano peptide, gets released within PVN to modulate the HPA axis.It is supposed that stress induced oxytocin release from magnocellular neurons of PVN is mediated by the binding of CRH to CRF2 receptors (CRF, corticotropin releasing factor, an alternate term for CRH) on oxytocinergic neurons of PVN, as well by the binding of corticosteroid released by the adrenal glands [7]. Oxytocin thus released binds to its receptors on wide range of neural structures in the brain including the CRH producing cells of PVN with increased activity during stress.

It is hypothesized that oxytocin down regulates CRH expression by regulating the translocation of CRTC. Oxytocin is observed to inhibit CRTC3 translocation and thus delay stress-induced CRHgene transcription in the PVN probably through the mediation of salt-inducible kinase (SIK) intracellular signalling pathway that couples the oxytocin receptor to CRTC3. SIK is a major protein kinase known to regulate CRTC3/TORC3 (transducer of regulated cyclic AMP-response element-binding protein 3) nuclear trafficking. SIK which is a member of the mammalian AMP-activated protein kinase (AMPK) family exists in hypothalamic neurons of the PVN in two isoforms (SIK1 & SIK2) and is known to inhibit CRHtranscription by impairing CRTC trafficking to the nucleus. Thus oxytocin exerts an inhibitory effect on stress axis activation by impairing the binding of CREB to the promoter region of CRH gene and thus impedes with the transcription of the hormone [8].

The oxytocinergic system is not confined to PVN alone but is also found to involve various forebrain areas like hippocampus, amygdala, bed nuclei of the stria terminalis (BNST), ventrolateral septum (LSV), and several hypothalamic nuclei as these structures are found to express oxytocin receptors on their surfaces [9]. It is observed that many of these structures could exert an inhibitory modulation over HPA axis by their GABAergic efferents to PVN, thus inferring the existence of integrated inhibitory pathway of stress attenuation [3]. Oxytocin released within the amygdala reduces the reactivity to fear and stress and consequently LC activity is decreased whereby noradrenergic stimulation of CRH release declines. Oxytocin released from the neurons that project from the PVN to the NTS activates alpha 2- adrenoreceptors, which inhibit the activity of the noradrenergic neurons in the LC and NTS. Oxytocin is also released in response to perceived stress in which case oxytocin release is paralleled with the stress pathway where it plays a role in dampening the stress responses and facilitates coping behaviours [6].

## Evidences for oxytocin’s stress attenuation effect:

The anxiolytic effect and stress suppressing efficiency of oxytocin is evident from various physiological conditions where oxytocinergic system is upregulated as in pregnancy, lactation, skin-to-skin contact between mothers and infantsetc. It has also been reported thatmassage, food intake, warm social interactions between individuals, friendly interaction with a beloved dog etc, can also upregulatethe central oxytocinergic pathway, enhancing emotional stability and decreasing the susceptibility to stress. Oxytocin enhancement is observed to improve social interactive behaviours by causing the release of serotonin in amygdala, insula, and hippocampus, and induces wellbeingby stimulating dopamine release in the nucleus accumbens. Oxytocin decreases sensitivity to pain by increasing opioidergic activity in the peri aqueductal gray (PAG) and has anti-stress effects with a long-term perspective linked to good mental and physical health and protection from certain stress related diseases [6].

It is hypothesised that high anxiety states result from a low oxytocin activity in brain, which could be either due a low hypothalamic expression of oxytocin gene, low levels of central oxytocin release or a low availability of oxytocin in the local extra cellular fluid, impaired expression of oxytocin receptor or an insignificant binding of oxytocin to the regions of the brain concerned with emotional and social behaviours. Therapeutic trials with oxytocin administration in various stress related psychopathologies likeposttraumatic stress disorder, generalized anxiety disorder, social anxiety disorder, autism and schizophrenia, where oxytocinergic system is observed to bedysregulated due to genetic or epigenetic mechanisms, makes the inference that oxytocinis a potential candidate to attenuate stress as post interventional serum cortisol levels was observed to be reduced [10].

## Conclusion:

Oxytocin, the hypothalamic neuro-peptide that mediates many physiological and behavioural functions, is a potent stress attenuating neuro modulator as it down regulates stress by impairing the transcription of CRH gene that initiates stress axis.
